# Integrative Transcriptome and Metabolome Analysis Reveals the Regulatory Networks and Key Biosynthetic Pathway Genes of Wild and Cultivated *Gentiana macrophylla* Pall

**DOI:** 10.3390/metabo16030184

**Published:** 2026-03-10

**Authors:** Juanjuan Liu, Jialing Zhang, Yiyang Chen, Ke Li, Xiaohui Ma, Xiaobo Zhang, Ling Jin

**Affiliations:** 1 School of Pharmacy, Gansu University of Traditional Chinese Medicine, Lanzhou 730000, China; gszyljj2023@163.com (J.L.); zhjl3615@163.com (J.Z.); chenyy001012@163.com (Y.C.); ke.lee@outlook.com (K.L.); zyxymxh@163.com (X.M.); 2 State Key Laboratory for Quality Ensurance and Sustainable Use of Dao-di Herbs, National Resource Center for Chinese Materia Medica, China Academy of Chinese Medical Sciences, Beijing 100700, China; 3 School of Pharmacy, Nanjing University of Traditional Chinese Medicine, Nanjing 210023, China; 4 Engineering Research Center for Evaluation, Protection, and Utilization of Rare Traditional Chinese Medicine Resources, Lanzhou 730000, China; 5 Gansu Pharmaceutical Industry Innovation Research Institute, Lanzhou 730000, China; 6 Northwest Collaborative Innovation Center for Traditional Chinese Medicine Co-Constructed by Gansu Province & MOE of PRC, Lanzhou 730000, China

**Keywords:** *Gentiana macrophylla* (Gentianaceae), wild, cultivated, multi-omics, Iridoids, medicinal value

## Abstract

Objectives: *Gentiana macrophylla* Pall. (Gentianaceae) is a medicinally important perennial herb. Iridoids are the main chemical constituents of *G. macrophylla.* The scarcity of the wild resource has led to increased attention to cultivated *G. macrophylla*. However, little is known about the metabolic differences and the regulatory mechanisms between cultivated and wild *G. macrophylla*. Methods: This study utilized untargeted metabolomics and transcriptomics to reveal differentially accumulated metabolites (DAMs) and differentially expressed genes (DEGs) between wild and cultivated. Results: The metabolomics profiling revealed 25,587 DAMs (*p* < 0.05) while the transcriptomic profiling identified 6830 DEGs. Analysis revealed that DEGs were predominantly enriched for processes associated with monoterpenoid biosynthesis and flavonoid biosynthesis. In addition, we word validated six DEGs involved in monoterpenoid biosynthesis and flavonoid biosynthesis by RT-qPCR. According to KEGG pathway analysis, *10HGO* (8-hydroxygeraniol dehydrogenase) may be a key enzyme encoding secoiridoid biosynthesis. The comprehensive results of transcriptome and metabolomics analysis revealed significant correlation between metabolite content and gene expression, providing a foundation for further study the function of *G. macrophylla* Pall. and the regulation of biosynthesis of active components. Conclusions: These approaches aim to explore the consistency of medicinal quality between the two sources across different habitats and to develop cultivated gentian as a full substitute for its wild counterpart in medicinal value. This strategy will fundamentally alleviate the predatory harvesting pressure on wild resources, ease their depletion, provide a theoretical basis for further development and protection of wild species of *G. macrophylla* in the future.

## 1. Introduction

*Gentiana macrophylla* (Gentianaceae) is a traditional medicinal plant, which was first recorded in *the Shennong’s Herbal Classic*. The *Chinese Pharmacopoeia* stipulates that the medicinal part of *Gentiana macrophylla* Pall., *Gentiana straminea* Maxim., *Gentiana crassicaulis* Duthie ex Burk., and *Gentiana dahurica* Fisch. are dry root, which is mainly used to treat rheumatism and arthralgia, damp heat jaundice, bone steaming hot flashes, and infantile malnutrition fever [1]. It has the effects of dispelling rheumatism, relieving arthralgia, and relieving deficiency heat. Modern pharmacological studies have shown that *G. macrophylla* has anti-inflammatory, analgesic, antibacterial, antiviral, anti-tumor, immune regulation, liver protection, antioxidant, and other pharmacological activities [2,3,4,5]. The main chemical constituents include iridoids, triterpenoids, lignans, flavonoids, alkaloids, steroids, polysaccharides, and other types. Among them, iridoids are the main chemical constituents of *G. macrophylla*, belonging to monoterpenes [6].

The market survey found that due to the mixed origin of *G. macrophylla*, the resources of wild *G. macrophylla* Pall. have been reduced year by year due to long-term over-harvesting; this has resulted in a diminished market presence for this species, which is further complicated by its mixed genetic origins. The producing areas are mostly concentrated in Ningxia, Shaanxi, and Shanxi in China. The cultivated *G. macrophylla* holds a large market share in Shaanxi Province, China. The chemical composition, quality, and genetic diversity of medicinal materials are known to be influenced by growth environment and cultivation practices [6,7,8,9]. Although differences in morphology and certain chemical components between wild and cultivated *G. macrophylla* have been reported [10], comparative studies at the molecular level remain scarce. These quality and efficacy variations lead to significant price fluctuations between wild and cultivated materials, highlighting the importance of standardized cultivation. The therapeutic effects of *G. macrophylla* are attributed to its complex metabolite profile. However, systematic investigations into the differences in integrated metabolic profiles and the underlying molecular mechanisms governing metabolic diversity between wild and cultivated *G. macrophylla* are still limited. However, part of the content involves exploring relevant studies in the Gentianaceae family [11,12].

The basic root shapes are similar, but the cultivated *G. macrophylla* is significantly thicker, with a paler surface, a denser root head, and more branches in the lower part. Microscopically, they are largely alike, the main difference being more xylem bundles in the root head of the cultivated variety [13]. Seven constituents, such as loganic acid, sweroside, 6’-O-β-D-glucosyl gentiopicroside and swertiamarin, were identified as biomarkers representing major differences between the two varieties [14].

Metabolomics is a powerful means to the analysis and identification of the overall metabolic profiles in an organism. It is widely used in a variety of plants and Chinese herbal medicines. High-throughput transcriptome sequencing provides solutions for the discovery of functional genes associated with primary and secondary metabolites biosynthesis [15]. Currently, techniques combining high-throughput metabolomics and RNA-sequencing (RNA-seq) technologies have been developed to study the dramatic changes of various primary and secondary metabolites in plants and Chinese herbs, and reveal the DEGs associated with biosynthesis of metabolites [16]. In this study, metabolic differences and associated gene expression between cultivated and wild types were investigated using a strategy integrating non-targeted metabolomics and transcriptomics [11]. The study not only helps to better understand the molecular differences between wild and cultivated *G. macrophylla* Pall but also provides a reliable basis for the quality research of cultivated *G. macrophylla* Pall [17].

## 2. Materials and Methods

### 2.1. Chemicals and Reagents

HPLC-grade acetonitrile and methanol were purchased from CNW Technologies. Ammonium acetate was purchased from Sigma-Aldrich, Inc. (St. Louis, MO, USA). Ethanoic acid was purchased from Fisher Chemical (Shanghai Civi Chemical Technology Co., Ltd., Shanghai, China), and ddH_2_O is Watsons (Shanghai Civi Chemical Technology Co., Ltd., Shanghai, China). β-mercaptoethanol was purchased from Xilong Science (Xilong Science Co., Ltd., Shantou, China).

### 2.2. Sample Collection

Regarding the cultivation samples, they were propagated from seeds. The wild and cultivated specimens of *G. macrophylla* were ensured to be at the mature, medicinal stage, and were specifically procured from the Qinling region, a location renowned for its rich biodiversity and traditional medicinal plant cultivation. The climatic conditions are largely consistent, thereby minimizing the influence of geographical variability. A total of 10 wild samples were collected and 10 cultivated. The original plant and plant roots were identified as *G. macrophylla* Pall. in the gentian family by Professor Jin Ling of the School of Pharmacy, Gansu University of Traditional Chinese Medicine. Voucher specimens (621202130820006LY) were deposited in the plant herbarium of Pharmaceutical College, Gansu University of Traditional Chinese Medicine, PR China. Wild and cultivated roots were mixed and divided into 3 groups as 3 biological replicates. The fresh material was cut directly into small pieces, immediately frozen in liquid nitrogen and stored at −80 °C until further transcriptomic and metabolomic analysis.

### 2.3. UHPLC-QE-MS Non-Target Metabolomics Analysis

#### 2.3.1. Extraction and Separation of Metabolites

The samples were subjected to a freeze-drying process to remove moisture while they were meticulously ground into a fine powder, ensuring a uniform consistency that is conducive to the subsequent analytical procedures. Weigh each sample (20 mg) into an Eppendorf tube and add 1000 μL of the extraction solution (methanol–acetonitrile–water = 2:2:1, with the isotopically labeled internal standard mixture) [18]. The sample was then homogenized for 4 min using a homogenizer with a frequency of 35 Hz, followed by ultrasound in an ice water bath for 5 min. This combined process of homogenization and sonication was performed in three cycles to ensure thorough extraction. Subsequently, the samples were incubated at −40 °C for 1 h and centrifuged at 4 °C for 15 min at 12,000 rpm (RCF = 13,800× *g*), R = 8.6 cm). The supernatant containing the extracted component was carefully filtered through a 0.22 μm microporous filter membrane and transferred to a glass vial for further analysis [19].

Ultra-high-performance liquid chromatography coupled with quadrupole-Orbitrap mass spectrometry (UHPLC-QE-MS) (Thermo Fisher Scientific, Waltham, MA, USA) was employed for non-targeted metabolomics analysis. It uses a UHPLC system (Vanquish, Thermo Fisher Scientific) with a Phenomenex Kinetex C18 (2.1 mm × 100 mm, 2.6 μm) coupled to Orbitrap Exploris 120 mass spectrometer (Orbitrap MS, Thermo). To ensure data quality and reproducibility, a pooled quality control (QC) sample was generated by combining equal volumes from each experimental sample. The QC sample was injected at the beginning of the analytical sequence to condition the system and then repeatedly after every 10 experimental samples to monitor instrument stability throughout the run.

The mobile phase consisted of 0.01% acetic acid aqueous solution (A) and isopropanol/acetonitrile (1:1, *v*/*v*) (B). The temperature of the automatic sampler was 4 °C, and the injection volume was 2 μL [20]. The orbital well Exploris 120 mass spectrometer was used to acquire MS/MS spectra in information-dependent acquisition (IDA) mode under the control of acquisition software (Excalibur, Thermo Corporation, Waltham, MA, USA, v1.0). In this mode, the acquisition software continuously evaluates the full-scan MS spectrum. The ESI source conditions were set as follows: sheath gas flow rate as 50 Arb, Aux gas flow rate as 15 Arb, capillary temperature 320 °C, full MS resolution as 60,000, MS/MS resolution as 15,000 collision energy as 20/30/40 in SNCE mode, spray Voltage as 3.8 kV (positive) or −3.4 kV (negative), respectively [21,22].

#### 2.3.2. Pre-Identification and Analysis of Metabolites

ProteoWizard converts raw data into mzXML format for peak detection, extraction, alignment, and integration. [23]. The internal MS2 database (BiotreeDB) was then used for metabolite annotation. The threshold for comments is set to 0.3. Multivariate data analysis, including principal component analysis (PCA) and orthogonal partial least squares discriminant analysis (OPLS-DA, *n* = 200 permutations), was performed on the processed data and adjusted to the total peak intensity.

### 2.4. RNAseq-Based Transcriptome Analysis

The experimental process of transcriptome sequencing includes RNA extraction, RNA detection, library construction, and computer sequencing. Total RNA for 3 biological repeats for RNAseq was extracted from the root, with about 1 μg RNA per sample as input material for RNA sample preparation. The purity, concentration, and integrity of RNA were detected by NanoDrop 2000 spectrophotometer (NanoDrop™ 2000; Thermo Fisher Scientific). Sequencing libraries were generated using NEBNext^®^Ultra™ RNA Library Prep Kit for Illumina^®^ (NEB, San Diego, CA, USA, v2.14.0) and index codes were added to attribute sequences to each sample. To control for potential contamination, negative controls (non-template controls) were included during both the RNA extraction and cDNA library construction steps. Raw reads were processed to remove adapter sequences and low-quality reads. Specifically, reads were discarded if they contained >10% ambiguous nucleotides (N) or if more than 50% of their bases having Q ≤ 10. The resulting high-quality clean data were provided in FASTQ format.

Cluster the indexed encoded samples on the cBot Cluster Generation System using the TruSeq PE Cluster Kit V3-Cbott-HS (Illumina, San Diego, CA, USA, v2.1). After cluster generation, the library was prepared for sequencing and double-terminal reads were generated. The sequences were further processed using the online platform of Baimai Cloud (www.biocloud.net, accessed on 28 April 2024).). Clean reads were obtained by removing reads containing joints, reads containing poly-N and low-quality reads from the original data. At the same time, Q20, Q30, base composition and sequence repetition level of clean data were calculated. All downstream analysis is based on high-quality, clean data.

Unigene N50 is defined as the length of the longest unigene such that all unigenes of that length or longer account for at least 50% of the total length of all unigenes, when unigenes are sorted from longest to shortest and their lengths are cumulatively summed. In this project, a total of 64,601 unigenes were assembled, with an N50 of 1756 bp, indicating high assembly completeness. Detailed assembly statistics are provided in Supplementary Table S1.

The DESeq2R package (1.10.1) was used for differential expression analysis between the two groups. DESeq provides statistical routines for determining differential expression in digital gene expression data using a model based on negative binomial distribution. The *p*-values of the results were adjusted using Benjamini and Hochberg’s method to control for error discovery rates. Genes with an adjusted *p*-value < 0.05 found by DESeq were assigned as differentially expressed. (v1.34.0, fold-change ≥ 2, correction *p* value ≤ 0.05). A clustering heatmap presents the results of both functional annotation/enrichment analysis for DEGs and the profiling of novel genes [24].

### 2.5. Integrated Analysis Between Metabolite Profiling and Transcriptome

The results of differentially expressed metabolite (metabolome) analysis were combined with the results of transcriptome analysis [25]. The genes showing altered transcriptomic, as well as metabolomic profiles, were mapped to the KEGG pathway map, showing pathways of simultaneous enrichment of DAMs and DEGs [26]. The correlation between DEGs and metabolites in each group was analyzed and the network diagram was drawn. The correlation between them was then analyzed [25].

### 2.6. Validation of Key DEG with Quantitative Real-Time PCR

To verify the expression profile of DEGs obtained by RNA-seq, by screening the genes involved in the biosynthesis pathway of monoterpenoid and flavonoids one by one, 6 key DEGs were obtained and word validated by real-time RT-qPCR. With TRINITY_DN14216_c2_g1_i1 as the internal reference gene, primers were designed according to the required sequence of the target gene (Table 1). Using EASYspin Plus Polyphenols/Complex Plant RNA Rapid Extraction Kit (Aidlab, Beijing, China), RT-q PCR (Guangzhou Hehui Biotechnology Co., Ltd., Guangzhou, China, H-9800) was performed for each tissue sample of the collected plant material with 3 biological replicates and at least 3 technical replicates per biological replicate [27]. cDNA was obtained using a reverse transcription kit (TransGen Biotech Co., Ltd., Beijing, China). PCR amplification was performed using a 2X M5 UItraSYBR Mixture (Low ROX), which were from Thermo Fisher Scientific (2720 Thermal Cycler). Each reaction volume was 16 μL, including 2 μL cDNA, 8 μL of 2 × M5 HiPer SYBR ^®^ Premix Es Taq TM, 0.32 μL upstream and downstream primers (10 umol·L^−1^), and 5.36 μL of ddH_2_O. The thermal cycling profile was: 95 °C for 10 min (hot-start activation) followed by 40 cycles of 95 °C for 10 s (denaturation), 60 °C for 30 s (annealing), and 72 °C for 20 s (extension). The relative expression was calculated by the 2^−ΔΔCT^ method. The expression levels of 6 genes were consistent with RNA-seq data. This indicates that the sampling method and RNA-seq used in this study are suitable for studying the transcriptome model of *G. macrophylla* TRs.

### 2.7. Statistical Analysis

Microbioinformatics (online bioinformatics analysis, visualization cloud platform) and chiplot online platform were used to map DAMs and differential gene data, such as cluster heatmap, volcano map, and matchstick map. SIMCA 14.1 software was used to draw the metabolite PCA and OPLS-DA maps. GraphPad Prism 8 was used to visualize the significance of the combined analysis of DAMs and DEGs. SPSS Statistics 26 software was used to analyze whether the data were statistically significant (an alpha value of *p* < 0.05 was considered statistically significant).

### 2.8. Integration of Metabolomic and Transcriptomic Data

To establish functional links between metabolic and transcriptional changes, we performed an integrated correlation analysis focused on KEGG pathways co-enriched by both DAMs and DEGs. For each co-enriched pathway, the abundance of every DAM was pairwise-correlated with the normalized expression of every DEG within that pathway across all biological samples, using Pearson’s correlation. A stringent dual threshold was applied to define significant associations: an absolute correlation coefficient |r| > 0.8 and a significance level of *p*-value < 0.05 (FDR-adjusted). Metabolite–gene pairs passing these thresholds were considered for further analysis. The final selection of key metabolites (n = 37) and genes (n = 6) for detailed discussion was based on a combination of: (1) the strength and significance of their correlation, (2) their known biological importance as major bioactive compounds or pathway enzymes in *G. macrophylla*, and (3) their central position within the enriched pathways.

## 3. Results

### 3.1. Metabolite Profiling of Samples

#### 3.1.1. Multivariate Statistical Analysis of Metabolites

UHPLC-QE-MS non-target metabolites analysis was performed to determine metabolic profiles of *G. macrophylla* Pall. root tissues. Based on the qualitative and quantitative metabolomics results of the UHPLC-QE-MS, univariate statistical analysis and multivariate statistical analysis were carried out (MVA) to screen for metabolites with significant differences (VIP > 1 and *p*-value < 0.05) [28], among which shikimate pathway derivatives, shikimates and phenylpropanoids (25.51%), terpenoids (10.38%), and fatty acids (6.42%) are the main metabolites. They included flavonoids (132), iridoids monoterpenoids (32), secoiridoid monoterpenoids (10), lignans (28), and coumarins (47).

**Figure 1:**
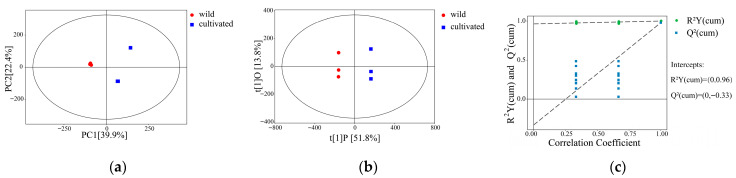
(**a**) PCA plot of DAMs. (**b**) OPLS-DA plot of DAMs. (**c**) Permutation plot test of OPLS-DA model.

Multivariate statistical analysis methods, such as PCA and OPLS-DA models, were inserted into the metabolite list after processing for further analysis to reduce the data dimension and improve the interpretability and validity of the data. All the samples in the PCA score plots were in the 95% confidence ellipse, and the proportion of two principal components was 62.3%. The coefficient of variation of the first principal component was 39.9%, and the coefficient of variation of the second principal component was 22.4%, indicating the metabolites of wild and cultivated *G. macrophylla.* Pall (Figure 1a). PCA algorithms based on visual discrimination generally cannot account for the exact differences between samples. In this study, we used the OPLS-DA model for supervised classification to measure the degree of metabolite conversion in significantly separated samples of different wild and cultivated *G. macrophylla*. Figure 1b,c show a high degree of differentiation between the sample groups in the OPLS-DA plots, with significant differences between the different groups. Our experiments also reveal excellent model parameters. Through cross-validation and response replacement test (RPT), OPLS-DA model does not appear overfitting (R^2^Y = (0, 0.96), Q^2^ = (0, −0.33)). Based on these findings, OPLS-DA was proved to be an effective prediction model for analyzing metabolic differences between wild and cultivated *G. macrophylla* Pall.

#### 3.1.2. Screening and Classification of the DAMs

**Figure 2:**
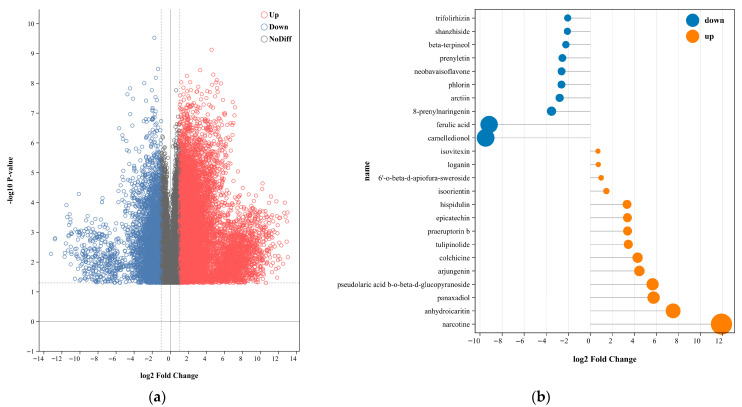
(**a**) Volcano plot of metabolites in wild vs. cultivated *G. macrophylla*. (**b**) Matchstick analysis of metabolites in wild vs. cultivated *G. macrophylla*.

In the screening process, *p-*value < 0.05 and FC (fold change) ≥ 2 or FC (fold change) < 0.5 were used as screening criteria. There were a total of 60,935 metabolite features. Of the 60,935 metabolite features detected, 19,228 (31.55%) were confidently identified at MSI Level 1 (authentic standards), and an additional 1620 (2.66%) were putatively annotated at Level 2. The vast majority (65.79%) remain unidentified and were retained as unknown features for differential analysis. A volcano plot was utilized to visualize the results of 25,587 DAMs (as depicted in Figure 2a). Compared with cultivated *G. macrophylla*, a total of 15,991 metabolites were up-regulated (expressed in red) and 9596 metabolites were down-regulated (expressed in blue) in wild *G. macrophylla*. Metabolites that did not show significant changes were represented in gray. A thorough analysis was performed to identify the top differentially expressed metabolites, both up-regulated and down-regulated. The top 10 metabolites in each category were narcotine, camelledionol, ferulic acid, anhydroicaritin, panaxadiol, pseudolaric acid b-o-beta-d-glucopyranoside, arjungenin, colchicine, 8-prenylnaringenin, tulipinolide, as shown in the matchstick analysis (Figure 2b). According to the mean value of relative quantitative metabolites, cultivated *G. macrophylla* was 0.809, wild *G. macrophylla* was 0.737, and cultivated was higher than wild. Flavonoids, phenolic acids, and lignans were higher in cultivation than in the wild, while isoflavonoids, stilbenoids, and coumarins were higher in the wild than in cultivation. Wild terpenoid metabolites were higher than cultivated ones, and the difference in iridoids monoterpenoids’ metabolism was the same as that of cultivated ones, with 36 species up-regulated and 39 species down-regulated, among which iridoids monoterpenoids were up-regulated and 6 species down-regulated. The number of wild fatty acids was higher than that of cultivated fatty acids, and the number of up-regulated (17) and down-regulated (18) fatty acids was higher than that of cultivated fatty acids.

#### 3.1.3. Metabolic Pathway Enrichment Analysis of DAMs Between Wild and Cultivated *G. macrophylla*

**Figure 3:**
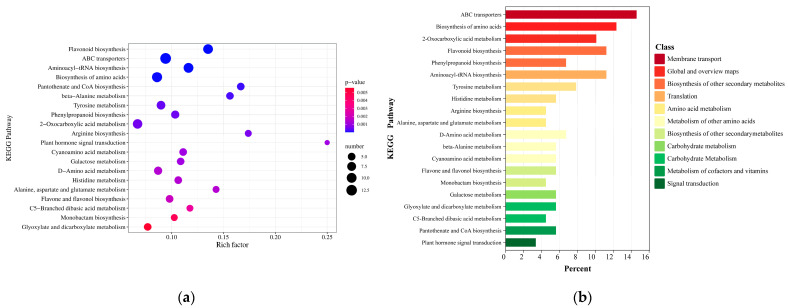
(**a**) Enrichment analysis of metabolic pathways based on DAMs in *G. macrophylla.* (**b**) KEGG classification diagram of DAMs in G. macrophylla.

Metabolic pathway enrichment analysis can provide insight into the mechanisms of metabolic changes in different experimental samples [29]. The KEGG (www.genome.jp/kegg/, accessed on 26 April 2024) pathways of *G. macrophylla* Pall. can be divided into cellular processes, environmental information processing, genetic information processing, metabolism, and organic systems, which we mapped to 78 KEGG pathways [30]. When comparing wild and cultivated *G. macrophylla*, the enrichment of the top 20 differential metabolic pathways was performed, and the Rich Factor (the ratio of the number of DAMs annotated in one pathway to the number of all metabolites in the pathway) was calculated according to the enrichment results of the DAMs in the KEGG metabolic pathway [31]. The greater the value, the greater the degree of enrichment (Figure 3a). Metabolic pathways with significant enrichment of metabolites include glyoxylate and dicarboxylate metabolism, C5-branched dibasic acid metabolism, flavone, and flavonol biosynthesis, alanine, aspartate and glutamate metabolism, histidine metabolism, D−amino acid metabolism, galactose metabolism, cyanoamino acid metabolism, plant hormone signal transduction, arginine biosynthesis, 2−Oxocarboxylic acid metabolism, phenylpropanoid biosynthesis, tyrosine metabolism, beta−alanine metabolism, pantothenate and CoA biosynthesis, biosynthesis of amino acids, aminoacyl−tRNA biosynthesis, ABC transporters, and flavonoid biosynthesis. It is mainly subdivided into 11 categories (Figure 3b). The flavonoid biosynthesis and terpenoid backbone biosynthesis pathways exhibited high enrichment factors and involved a substantial number of metabolites, encompassing key metabolites such as luteolin, gentiopicroside, and loganic acid.

### 3.2. Transcriptome Analysis of Wild and Cultivated Samples of G. macrophylla

#### 3.2.1. Annotation and Expression of the Unigenes

**Figure 4:**
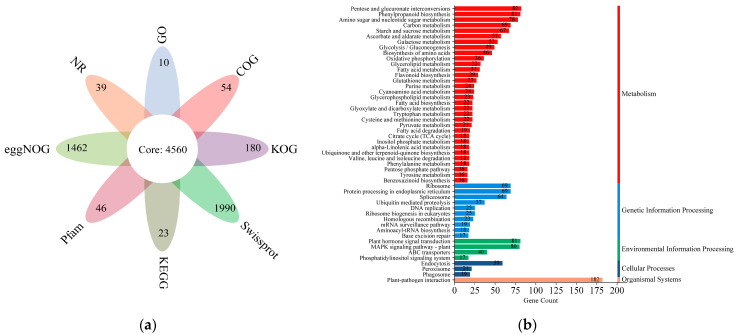
(**a**) All database petal figure (each petal represents an annotated gene unique to the database, and the flower center represents an annotated gene shared by all databases). (**b**) KEGG annotated genes.

A transcriptomic dataset of the cultivated and wild *G. macrophylla* Pall. was constructed and the results were summarized [27]. The transcriptome sequencing of 6 samples was completed, and a total of 43.82 Gb of Clean Data was obtained, with the Clean Data of each sample reaching 6.56 Gb, and the percentage of Q30 base was 93.17% or above. A total of 64,601 Unigene were obtained after assembly. Among them, there are 19,362 Unigene with a length of more than 1kb. A total of 31,722 Unigene annotation results were obtained, All the unigene assemblies were annotated into eight public databases, including nonredundant protein sequences (NR), Swiss-Prot (www.sib.swiss/swiss-prot/, accessed on 26 April 2024), Pfam (http://pfam.xfam.org/, accessed on 26 April 2024), Clusters of Orthologous Groups (COG, http://www.ncbi.nlm.nih.gov/COG/, accessed on 26 April 2024), Gene Ontology (GO, https://geneontology.org/, accessed on 26 April 2024), and the Kyoto Encyclopedia of Genes and Genomes (KEGG), and euKaryotic Ortholog Groups (KOG), and evolutionary genealogy of genes (eggNOG). NR is the most annotated database, with 30,652 unigenes, accounting for 96.63% of the total number of unigenes. The GO, KEGG, COG, Swiss-Prot, Pfam, KOG, and eggNOG, databases annotated 24,691 (77.83%), 20,036 (63.16%), 7519 (23.70%), 19,176 (60.45%), 20,413 (64.34%), 16,918 (53.33%), and 24.750 (78.02%) unigenes, respectively. All database petal figures (Figure 4a).

The GO enrichment analysis revealed that the DEGs were primarily clustered into three major categories: cellular component, molecular function, and biological process. KEGG database was used to annotate unigenes to further elucidate their role in biosynthesis and metabolic pathways. These unigenes were categorized into five main groups, which encompassed 50 sub-clusters. The largest number of KEGG identifiers was in plant–pathogen interaction (182 annotated genes), followed by pentose and glucuronate interconversions (82 annotated genes), plant hormone signal transduction, and phenylpropanoid biosynthesis (81 annotated genes) (Figure 4b).

#### 3.2.2. Functional Analysis of DEGs

**Figure 5:**
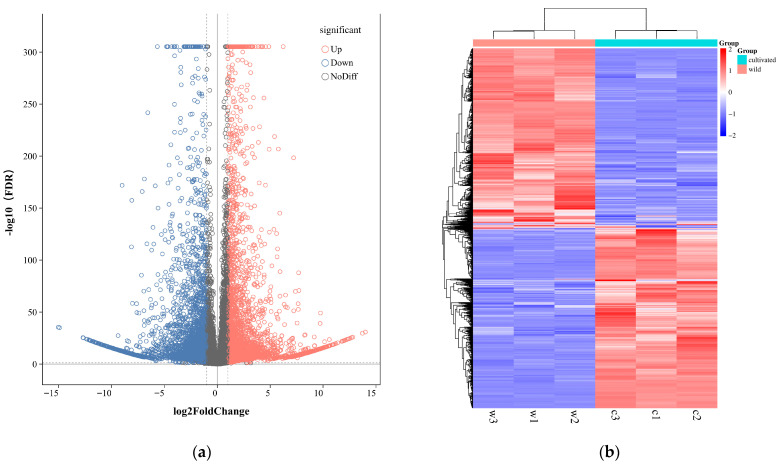
(**a**) Differentially expressed gene volcano map in wild vs. cultivated *G. macrophylla*. (**b**) Heatmap of differential gene clustering in wild vs. cultivated *G. macrophylla*.

The genes with significantly different expression levels in different samples are called DEGs, and the annotation and analysis of the metabolic pathway of DEGs will help to further understand the function of the genes. In the screening process, FDR < 0.01 and FC (fold change) ≥ 2 were used as screening criteria. Because gene expression is spatiotemporal specific, we compared the transcriptome data from two different growth modes. Compared with cultivated *G. macrophylla*, a total of 3438 DEGs were up-regulated and 3392 DEGs were down-regulated in wild *G. macrophylla*. Red and blue indicate up-regulated and down-regulated genes, respectively (Figure 5a) [30]. The expression pattern of genes is closely related to their functions; genes with similar expression patterns are likely to have the same or related functions. In order to visually display the DEGs in different groups and mine new functional genes, hierarchical clustering analysis was performed on all DEGs screened. The genes with the same or similar expression patterns in different samples were clustered and displayed by heatmap. The figure shows a heatmap clustering the DEGs in wild and cultivated samples. Within the heatmap, deeper shades of red indicate higher gene expression levels in a given sample, while deeper shades of blue indicate lower expression levels. The top three most enriched terms among the up-regulated genes are: “pectin catabolic process”, “multi-organism process”, “response to stimulus”; the term with the highest proportion of down-regulated genes is “cellular process involved in reproduction in multicellular organism” (Figure 5b), and the potential functions of genes with similar expression patterns were discovered according to genes with known functions.

#### 3.2.3. Development of Simple Sequence Repeat (SSRs) Makers

Simple Sequence Repeats (SSRs) is one of the effective molecular markers for detecting genetic diversity and constructing genetic maps. The de novo transcriptome assembly used for SSRs discovery was constructed by pooling sequencing reads from both wild and cultivated samples, comprising a total of 10,861 unigenes. Through SSRs analysis, unigenes larger than 1 KB are screened by MISA software (v1.0).. The minimum number of repeats required for detection was defined per motif size: mononucleotide (10), dinucleotide (6), and trinucleotide to hexanucleotide (5 each). An interrupting distance of ≤100 bp was used to define compound SSRs; six types of SSRs were identified: (1) mononucleotide repeat SSRs 5719, (2) dinucleotide repeat SSRs 1660, (3) trinucleotide repeat SSRs 2463, (4) tetranucleotide repeat SSRs 148, (5) pentanucleotide repeat SSRs 60, and (6) hexanucleotide repeat SSRs 70. In addition, the analysis identified 706 compound repeat SSRs and 33 overlapping compound type SSRs. A total of 10861 SSRs were characterized, contributing to the genetic diversity and offering potential markers for further study [30]. The validated SSRs markers in this study effectively revealed a clear genetic divergence between wild and cultivated populations of *G.macrophylla.* This was evidenced by polymorphism analysis, which demonstrated that mononucleotide and trinucleotide repeat SSRs, in particular, serve as highly efficient molecular markers for distinguishing between the two groups.

We found that the overall density and motif type distribution of SSRs in *G. macrophylla* generally follow trends observed in many plant genomes, with mononucleotide repeats being the most frequent. SSRs were mapped to gene regions, with a focus on identifying those within genes involved in key biosynthetic pathways. This was followed by an integrated analysis of the differentially expressed gene set, aiming to elucidate the potential impact of these SSRs on metabolite accumulation. While comprehensive genome-wide SSRs data for other Gentiana species are currently unavailable for direct comparison, the detailed profile established here for *G.macrophylla* may serve as a valuable reference for future comparative studies.

### 3.3. Integrated Analysis of the Metabolome and Transcriptome

**Figure 6:**
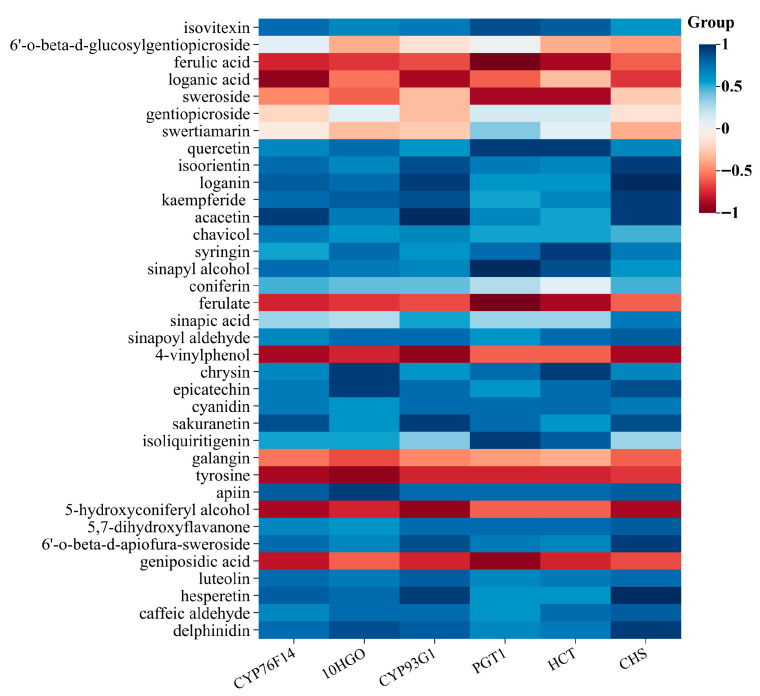
Correlation analysis of DEGs and DAMs.

Based on metabolomic and transcriptomics data, the KEGG pathway was selected as a vector and integrated analysis was performed. The KEGG analysis showed that the monoterpenoid biosynthesis (map00902) and flavonoid biosynthesis (map00941) pathways were significantly different between the two groups, and identical pathways were enriched in the transcriptome and metabolome. Several metabolites and genes that we focused upon were detected in these two pathways, involving a total of 37 metabolites and 6 DEGs (Figure 6). Integrated analysis of the metabolome and transcriptome is of great significance and provides references for mining key genes in the biosynthesis pathway of secondary metabolites.

**Figure 7:**
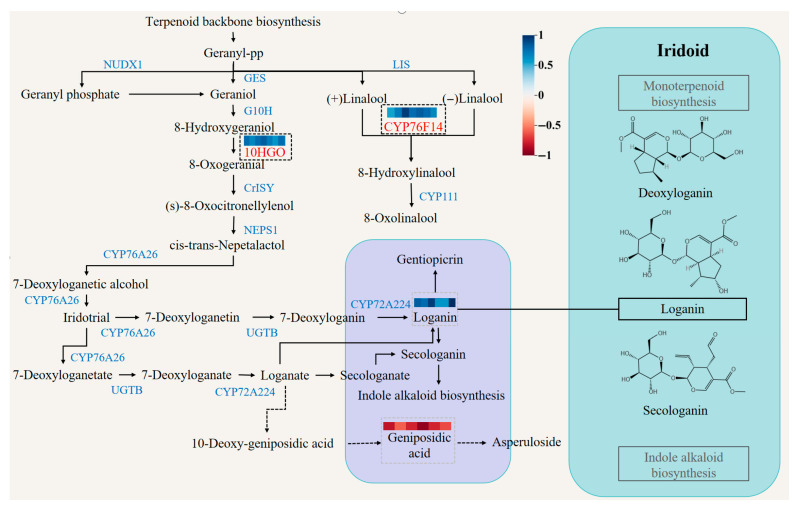
Overview of the terpenoid and monoterpenoid biosynthetic pathways in *G. macrophylla*.

Iridoids monoterpenoids and secoiridoid monoterpenoids are among the major active constituents of *G. macrophylla*. Therefore, we focused on the analysis and identification of the DEGs in the monoterpenoid pathway. Loganic acid had the highest correlation with *CYP76F14* ((E)-8-carboxylinalool synthase) (*r* = 0.94). Sweroside had the highest correlation with *PGT1* (*r* = 0.88) and *HCT* (*r* = 0.88) (shikimate O-hydroxycinnamoyltransferase). Gentiopicroside was negatively correlated with *CYP76F14*, *CYP93G1* (flavone synthase II) and *CHS* (chalcone synthase), but positively correlated with *10HGO*, *PGT1* (phlorizin synthase) and *HCT*. Swertiamarin was positively correlated with *PGT1* and *HCT*, but negatively correlated with other DEGs. Quercetin had the highest correlation with *PGT1* (*r* = 0.94). In the monoterpenoid pathway, *10HGO* encodes a well-characterized rate-limiting enzyme at the gateway to the iridoid skeleton and it is a critical transcriptional control point underlying iridoid production in cultivated *G. macrophylla* (Figure 7). Additionally, the relative expression levels of *10HGO* in both wild and cultivated samples were validated using qRT-PCR. The qRT-PCR results corroborated the expression patterns observed in the transcriptome data. Consequently, these findings hold significant value, providing insights and a reference framework for identifying and studying key genes involved in secondary metabolite biosynthesis pathways.

In addition to ferulate, 4-vinylphenol and galangin were negatively correlated, and the rest of the shikimates and phenylpropanoids were positively correlated. Among them, the correlation was highest between isovitexin and *PGT1* (*r* = 0.88). Isoorientin had the highest correlation with *CHS* (*r* = 0.94). Loganin and hesperetin had the highest correlation with *CHS* (*r* = 1). Sinapyl alcohol and ferulic acid had the highest correlation with *PGT1* (*r* = 1). Acacetin had the highest correlation with *CYP93G1* (*r* = 1). It suggests that these genes may be correlated with the biosynthesis of shikimates and phenylpropanoids, which are key pathways in the production of secondary metabolites. Additionally, the compound 6’-O-beta-D-glucosylgentiopicroside was found to have a positive correlation with the genes *CYP76F14* and *PGT1*, while showing a negative correlation with other genes.

To directly link transcriptional changes to the metabolic divergence between wild and cultivated *G. macrophylla*, we performed an integrated correlation analysis. “Flavonoid biosynthesis” was significantly associated with the expression of *HCT, CYP93G1, CHS*, and *PGT1.* Notably, *CHS* and *PGT1* exhibited significant up-regulation, concomitant with a marked increase in the levels of metabolites such as luteolin, epicatechin, and quercetin. Their involvement suggests a regulatory role in the expression of genes that contribute to the synthesis of flavonoids, which are significant for the medicinal attributes of the plant.

### 3.4. Word Validation of RNA-Seq Sequencing Data by qRT-PCR Analysis

**Figure 8:**
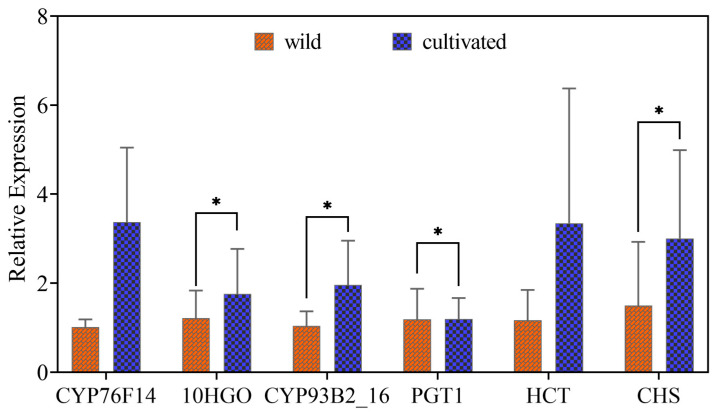
Word validation of RNA-Seq sequencing data by the qRT-PCR assay, * *p* ≤ 0.05.

The expression levels of 6 DEGs were consistent with the RNA-seq data (Figure 8). This suggests that both the sampling method and RNA-seq used in this study are suitable for studying transcriptome patterns of *G. macrophylla* Pall.

## 4. Discussion

Terpenoids (isoprenoids) are a class of important chemicals produced by plants. They have functions of anti-malaria, anti-tumor and anti-inflammation [32]. Terpenoids and isoprenoids play key roles in plant growth regulation and rhizosphere signaling, participate in the synthesis of diverse biomolecules, and contribute to plant defense against insects and microorganisms. These compounds also possess substantial medicinal value [33].

Iridoids are a special class of monoterpenes. Iridoids are a large class of active components or index components widely distributed in medicinal plants from *Gentianaceae* and other sources [34]. In the synthetic pathway, iridoids are intermediate products in the biosynthetic pathway of indole alkaloids [35]. Loganic acid and gentiopicroside are the products of monoterpenoid biosynthesis (map00902). Studies suggest that *10HGO* and *CYP76F14* play key roles in the biosynthesis of loganic acid and gentiopicroside. *10HGO* is a dehydrogenase involved in the biosynthesis of oxogeranial from hydroxygeraniol, a precursor of the terpenoid indole alkaloids. The significant up-regulation of loganic acid suggests that *10HGO* and *CYP76F14* may be the key genes responsible for its content differences between wild and cultivated *G. macrophylla.*

Although *HCT* is a key enzyme in the phenylpropanoid pathway and is primarily associated with lignin biosynthesis, its correlation with secoiridoid monoterpenoids likely reflects indirect metabolic coordination, as phenylpropanoid metabolism can influence carbon allocation and redox balance that also affect terpenoid biosynthesis, rather than a direct enzymatic role of *HCT. HCT* plays an important role in controlling phenylpropanoid flux and carbon allocation within secondary metabolism. Therefore, the observed correlation between *HCT* and secoiridoid monoterpenoids likely reflects system-level metabolic coordination and resource competition, rather than a direct biosynthetic connection.

In addition to terpenoids, flavonoids are also one of the main chemical constituents of *G. macrophylla*. At present, more than ten flavonoids and their glycosides have been isolated from this group of plants, which have antioxidant activity and strong antibacterial effect [36]. The parent nucleus of natural flavonoids is usually connected with substituents such as-OH, -OCH3 and isopentenyl, and is pale yellow to yellow [3]. The color of *G. macrophylla* may be closely related to flavonoids such as isovitexin and isoorientin. It is found that isovitexin has the highest correlation with *PGT1*, and isoorientin has the highest correlation with *CHS*.

We found that expression of *PGT1* in the flavonoid biosynthesis (map00941) was up-regulated significantly in wild *G. macrophylla* compared with that in cultivated *G. macrophylla. PGT1* is very important in plant growth, because *PGT1* may negatively regulate abscisic acid (ABA) signaling in guard cells and auxin-induced lateral root initiation [37]. Since the number of lateral roots of cultivated *G. macrophylla* was more than that of wild *G. macrophylla*, we speculated that *PGT1* regulated the growth of *G. macrophylla* roots. But no change in auxin inhibition of primary root growth was observed, suggesting that *PGT1* is specifically involved in negative regulation of auxin-induced lateral root initiation. However, this is insufficient to conclude the actual content of flavonoids. Of course, other hormones such as cytokinins, environmental signals, or post-translational regulation of PGT1 may act collectively to override the effect of its transcript level on lateral root phenotype in cultivated plants.

A joint analysis of DAMs and DEGs that 37 metabolites and 6 DEGs were involved, mainly involving the KEGG pathway for monoterpene biosynthesis (map00902) and flavonoid biosynthesis (map00941) pathway, which was word validated by RT-qPCR, and the results were consistent with RNA-seq data, and all are up-regulated. Through joint analysis, a comprehensive and systematic genetic information of *G. macrophylla* was obtained, which provided data support for the subsequent functional identification of *G. macrophylla* genes [38].

While our integrated metabolomic and transcriptomic approach provides valuable insights, several limitations should be acknowledged. First, the non-targeted metabolomics platform, although broad, may not capture all metabolites, particularly those at low abundance or lacking ionization efficiency. The correlative nature of our gene–metabolite association analysis identifies strong candidates but does not establish causality. Functional validation is required to confirm the regulatory roles of candidate genes. And, the genetic and metabolic differences observed may be influenced by local environmental factors and may not fully represent the variation across the entire geographical range of *G. macrophylla* or different cultivation practices.

## 5. Conclusions

Although previous studies reported few significant differences, our integrative analysis revealed multiple transcriptomic and metabolomic divergences between wild and cultivated *G. macrophylla*. In the joint analysis of DAMs and DEGs, we identified the DAMs (iridoid and flavonoid pathways) and DEGs (*CHS*, *PGT1, CYP93G1* and *10HGO*) of wild and cultivated *G. macrophylla.* That could guide the screening of germplasm resources and the breeding of superior *G. macrophylla* varieties. Moreover, the detailed annotation of secondary metabolite synthesis pathways provides essential insights for understanding the plant’s secondary metabolic processes. These findings highlight the critical need for the conservation of wild *G. macrophylla* species and thus provide a theoretical basis for future breeding and cultivation practices aimed at enhancing the quality and iridoid content of cultivated *G. macrophylla* species. It should be noted that the current findings are primarily correlative, and further functional validation of the candidate genes is essential to confirm their regulatory roles. Future research should also integrate multi-omics data from diverse populations and growth environments to establish a more comprehensive model for predicting and manipulating metabolite accumulation in *G. macrophylla.*

## Figures and Tables

**Table 1:** Primer sequence of candidate genes used for validation by real-time RT-qPCR.

Gene	Primer Sequence (5′—3′)	Expected Product Size (bp)
*CYP76F14*	Forward: AGTCATCGGGAAAGGCAAGG	145
	Reverse: TTCTTTGATGGCACACCGGA	
*10HGO*	Forward: CAGGGAGTGGTATTGGAGGG	120
	Reverse: GCTCCATTGCAGTGTTCACAT	
*CYP93G1*	Forward: ACGTTTCGTCTTCACCCTCC	139
	Reverse: ATCCAGCCACTTGGATGTCG	
*PGT1*	Forward: TGAAGTATGAAGACCGCCCG	155
	Reverse: ATACTTCCTTCACCGCTCCG	
*HCT*	Forward: GTAAAAACTCGGCCCAAGCG	119
	Reverse: CCCCCATAAGCAACCCCATT	
*CHS*	Forward: TCCGAACCGACTATCAACGC	110
	Reverse: CGTGGACCGAGTGAAACAGA	

## Data Availability

The datasets generated and/or analysed during the current study are available in the NCBI [National Center for Biotechnology Information: https://www.ncbi.nlm.nih.gov, accessed on 23 November 2025] repository, PRJNA1132749.

## Supplementary Materials

The following supporting information can be downloaded at: https://www.mdpi.com/article/10.3390/metabo16030184/s1, Table S1: Assembly Result Statistics Table; Table S2: SSR analysis summary statistics.
