# Supplementary material for: Integrative Transcriptome and Metabolome Analysis Reveals the Regulatory Networks and Key Biosynthetic Pathway Genes of Wild and Cultivated Gentiana macrophylla Pall
Source: Metabolites. 2026 Mar 10;16(3):184. doi: 10.3390/metabo16030184 (PMC13028276; doi:10.3390/metabo16030184)
Supplement: Supplementary file 1 [file metabolites-16-00184-s001.zip › metabolites-4124245-supplementary-done.pdf]

Table S1 Assembly Result Statistics Table

Length Range	Transcript	Unigene
200-300	22,516(15.74%)	16,652
300-500	27,850(19.47%)	15,358
500-1000	34,157(23.88%)	13,229
1000-2000	34,970(24.45%)	10,588
2000+	23,553(16.47%)	8,774
Total Number	143,046	64,601
Total Length	162,135,938	61,514,326
N50 Length	1,790	1,756
Mean Length	1133.45	952.22

Table S2 SSR analysis summary statistics

#type	number
c	706
c*	33
p1	5719
p2	1660
p3	2463
p4	148
p5	62
p6	70
Total	10861
